# Increase in Fast Response Time of the Resistance-to-Voltage Converter When Monitoring the Cable Products’ Insulation Resistance

**DOI:** 10.3390/s21020368

**Published:** 2021-01-07

**Authors:** Nikolay I. Yermoshin, Evgeny V. Yakimov, Aleksandr E. Goldshtein, Dmitry A. Sednev

**Affiliations:** School of Non-Destructive Testing, National Research Tomsk Polytechnic University, 30 Lenin Avenue, 634050 Tomsk, Russia; shishkovka@mail.ru (E.V.Y.); algol@tpu.ru (A.E.G.); sednev@tpu.ru (D.A.S.)

**Keywords:** resistance-to-voltage converter, cable insulation, insulation resistance, capacitance, electrical resistance measurement, settling time

## Abstract

Theoretical and experimental studies were conducted to investigate the impact of the cable capacitance during measurements of insulation resistance on the fast response time of a resistance-to-voltage converter. From a comparison of the results of simulation with the data obtained during the experiments, it was determined that the dependence characteristics of the settling time of resistance under measurement on the capacitance are identical to the analogous characteristics of electronic components of the resistance-to-voltage converter. It was experimentally proven that using T-shaped feedback in the resistance-to-voltage converter during the cable insulation resistance measurements reduces the settling time of the data by 1–3 times in comparison with a classical feedback system. Furthermore, when using the optimal parameters, the settling time of the resistance-to-voltage converter with T-shaped feedback depends to a lesser degree on the capacitance of the object under control.

## 1. Introduction

Cables, cords, and wires represent the most demanded products used in radio engineering, electrical, and electronic equipment. The main characteristic of the quality evaluation of cable insulation is electrical insulation resistance [1,2].

**Figure 1:**
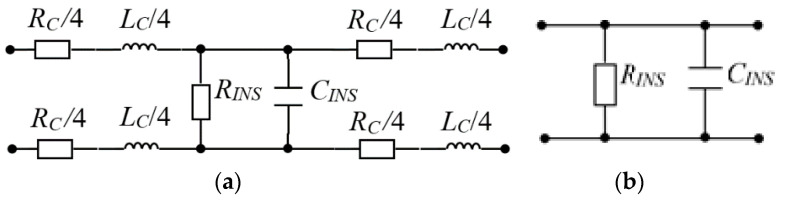
The circuit of real twin cable substitution: (**a**) equivalent; (**b**) simplified.

The complete circuit of cable substitution represents the chain with distributed parameters converted to the unit of length: conductor resistance *R_C_*, inductance *L_C_*, insulation capacitance *C_INS_*, and insulation resistance *R_INS_*. Figure 1a demonstrates the equivalent circuit of the cable substitution.

Inductance and conductor resistance of the cable produce impact only at high frequencies. Direct current or low frequencies are used during insulation resistance measurements. Due to this, the impact of inductance and conductor resistance is neglected during monitoring of insulation resistance of cables. For example, for coaxial cable RG6U [3] the following parameters are given: *R_C_* = 23.26 Ω/km, *R_INS_* = 1 GΩ·km, and *C_INS_* = 56.8 nF/km. The inductance value for cables is not given in the resources, however a formula exists for its calculation [1]:

(1) $$L_{C}=K+0.2\cdot \ln\frac{2S}{d},(\mathrm{mH}/\mathrm{km})$$

where *K*—constant, for cable RG6U equals 0.05 mH/km; *S*—axial spacing between conductors within the cable (mm), for cable RG6U equals 2.3 mm; *d*—the conductor diameter (mm), for cable RG6U equals 1.024 mm. 

It follows from Formula (1) that inductance of cable RG6U equals 0.35 mH/km. At a frequency of 50 Hz with a cable length of 1000 m, the total inductance resistance equals 0.11 Ω, total insulation capacity resistance is 56.1 kΩ, conductor resistance is 23.26 Ω, and insulation resistance is 1 GΩ. Thus, it has been observed that inductance reactance and conductor resistance are significantly less than insulation capacitance reactance and insulation resistance, respectively.

Figure 1b demonstrates the simplified circuit of real cable substitution with switched on in-parallel ideal capacitance and conductor resistance. Resistance *R_INS_* characterizes the resistive current component for all of the processes related to energy absorption.

The main problems of measurements of electrical cable insulation resistance pertain to [4,5,6]:

− the impact of the capacitance of cable products on the fast response time of measurement instruments (teraohmmeters);
− sensitivity of the teraohmmeter output chain to low frequency noise.

In compliance with the standard “UL 2556 Standard for Safety for Wire and Cable Test Methods” [7], the test voltage must be from 100 to 500 V. The value reading for electrical resistance of insulation during measurements is to be conducted after 1 min because of the momentum of applying the measuring voltage to the sample. The error of measurement should not exceed 10%.

Within the undertaken review, the methods for direct evaluation were considered. Their main advantage is the simplicity of device design and relatively high range of measurement. Among the limitations are low fast response time and the necessity of shielding the object under control to ensure the accuracy of resistance measurements [8,9,10].

The review of bridge techniques revealed that they are practically not used during monitoring of the cable products’ insulation resistance because they possess poor noise immunity and, as a sequence, have a high error of measurement [9,11,12,13].

Based on the Voltmeter–Ammeter method, a large number of monitoring devices have been developed. However, in most cases, the produced devices have a high measurement error, which either exceeds the acceptable set value [4,9,14] or reaches its boundary value. In addition, the limitations include a high reference voltage in the upper range of measurements, which does not satisfy the standard UL 2556 [7].

The method for charging and discharging capacitors possesses the best fast response time among other existing methods. A sufficient number of algorithms have been developed that enable the determination of the steady-state value of the resistance under measurement at the initial stage of charge (discharge) of the capacitive load. Fluke insulation testers are designed using this method. The best characteristics are possessed by Fluke 1555: the upper range of measurements is up to 2 TΩ, with an error of 20% at the reference voltage 10,000 V; the charging rate for capacitive load is 5 s/µF; and the discharge rate for capacitive load is 1.5 s/µF [15]. The limitations for the cable insulation resistance testers based on this method include the high error of measurement and high reference voltage, which are not in compliance with the standard applied for monitoring cable insulation resistance [7]. A high measurement error of signals results in poor noise immunity of monitoring devices.

Satisfactory noise immunity characterizes the instruments based on the method of converting resistance to voltage. The work [16] describes different variants of noise immune resistance-to-voltage converters, including a study of the converters’ fast response time. However, fast response time was assessed only with active resistance components. The measuring time for a resistor of 1 TΩ was 30 s. Work [17] describes a resistance-to-voltage converter in which the problem of shielding against low frequency noises was solved (less than 1 Hz).

Based on the review of the methods of measurement of cable products’ insulation resistance, the conclusion can be reached that teraohmmeters based on resistance-to-voltage converter possess the most efficient interference immunity. However, the resources do not contain sufficient information on studies of fast response time of resistance-to-voltage converters during measurements of cable insulation resistance, or on methods to increase their fast response time. Thus, the objective of this research is to study the feasibility of increasing fast response time of resistance-to-voltage converters during measurements of cable insulation resistance.

In this study, the fast response time of a resistance-to-voltage converter was compared with the classical feedback system and the fast response time of a resistance-to-voltage converter with T-shaped feedback during measurements of cable insulation resistance. First, simulation of resistance-to-voltage converter circuits was performed and then experimental testing was conducted.

## 2. Materials and Methods

If using an ideal operational amplifier, the output voltage of the resistance-to-voltage converter equals [18,19]:

(2) $$U_{OUT(ID)}=-U_{REF}\cdot \frac{R_{0}}{R_{INS}},$$

where *U_REF_*—DC reference voltage; *R_0_*—reference resistance.

From Formula (2), it is seen that the greater resistance *R_0_* in the feedback loop, the greater resistance can be measured. However, as a rule, the greater the resistor resistance, the lower its accuracy. Therefore, to ensure a small error of the resistance-to-voltage converter, the resistance of the feedback should be selected to have the lowest nominal value possible (less than 1 GΩ). For example, high voltage resistors from Riedon HVS Series within the range from 1 to 100 GΩ have an error of 1–2%, whereas within the range from 1 MΩ to 1 GΩ, the error is 0.25–0.5%, which is 4 times less [20].

**Figure 2:**
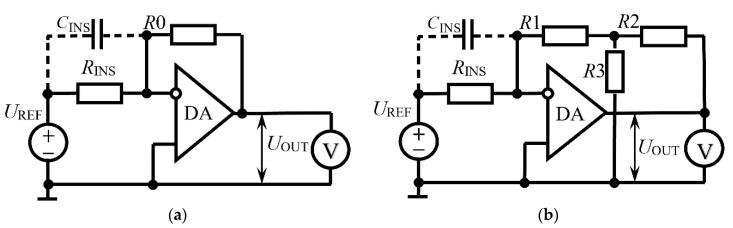
General scheme of the resistance-to-voltage converter (**a**) with a classical feedback system (**b**) with T-shaped feedback: *R_1_*, *R_2_* and *R_3_* are resistances of T-shaped feedback; *DA*—operational amplifier; *V*—voltmeter.

Using T-shaped feedback to obtain a stable useful signal requires resistors with lesser nominal characteristics, which theoretically allow reduction of the settling time for the output signal of the resistance-to-voltage converter and, accordingly, increase its fast response time. Figure 2 demonstrates the basic resistance-to-voltage converter.

**Figure 3:**
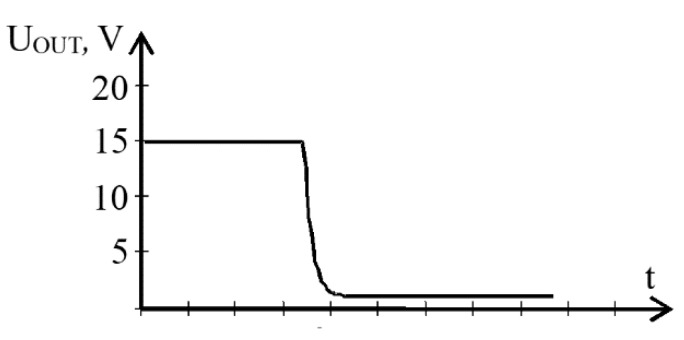
The transition process when switching on the teraohmmeter based on resistance-to-voltage converter.

In the case in which the operational amplifier does not possess a saturation mode, then, in the teraohmmeter designed based on the circuit presented in Figure 2, theoretically, the settling time of the data practically does not depend on the electrical capacitance of the cable because the object under study is under direct current voltage from the source with low output resistance *R_SOU_*. Under *R_SOU_* = 10 Ω and capacitance *C_INS_* = 56.8 nF, the time constant for the capacitor charge equals 568 × 10^–9^ s. A detailed examination of this circuit shows that a comparatively high capacitance *C_INS_* forms a differentiator amplifier. With the reference voltage placed on the resistance under study, high output voltage will tend to be formed on the converter output and the amplifier will transit to the saturation mode. The common mode is set during a long period of time, during which the cable capacitance charge occurs via reference resistance *R_0_* and input resistance of the operational amplifier. Figure 3 demonstrates the behavior of the transition process with the reference voltage placed on the resistance under study.

The transfer equation for the resistance-to-voltage converter with T-shaped feedback, using an ideal operational amplifier, is described with the expression [19,20]:

(3) $$U_{OUT(ID)}=-U_{REF}\cdot \frac{R_{1}+R_{2}+\frac{R_{1}\cdot R_{2}}{R_{3}}}{R_{INS}}=-U_{REF}\cdot \frac{R_{EQ}}{R_{INS}},$$

where *R_EQ_*—equivalent resistance of T-shaped feedback.

Equation (3) does not take into consideration many operational amplifier parameters (input voltage and current, zero offset voltage, target amplification coefficient, electrostatic protection, and others) which restrict the application of T-shaped feedback to the design of the resistance-to-voltage converter. In the work [20], using simulations and further proved in experiments, it was determined that the output voltage of the converter with T-shaped feedback *U_OUT_* does not depend on the combination of resistance *R*_2_ and *R*_3_, under the condition that they provide the same equivalent resistance *R_EQ_*. However, no explanation was provided for the useful signal bias of the output voltage caused by the significant decrease of the nominal resistance *R_1_* in relation to *R_EQ_*. Further research has revealed the decrease in the reference resistance *R*_1_ in relation to *R_EQ_* in multiples of increases in the voltage of the useful signal bias of the operational amplifier (*R*_3_ is too small in relation to *R_1_* and its value can be neglected). The statement is expressed by the following formula:

(4) $$U_{OUT.OF}=\frac{R_{EQ}}{\left(R_{1}+R_{3}\right)}\cdot U_{OF},$$

where *U_OUT_._OF_*—offset of the output voltage of the resistance-to-voltage converter with T-shaped feedback; *U_OF_*—offset voltage of the operational amplifier.

It is worth mentioning that Equation (4) does not consider all of the parameters of the operational amplifier, but only that which most significantly impacts the useful signal bias of the resistance-to-voltage converter.

To design the resistance-to-voltage converter with T-shaped feedback, it is necessary to use an operational amplifier with low offset voltage as the input operational amplifier. From a literature review [21,22], the most suitable variant of the operational amplifier is ADA4530-1, which combines low input current and low offset voltage (Table 1). This operational amplifier was used in all of the experiments.

Using T-shaped feedback with the ADA4530-1 operational amplifier allows the nominal reference resistance to be decreased by at least 100 times, which, with the useful signal in 1 V, results in an error in the signal offset of not more than 0.4%; thus, the offset voltage of the ADA4530-1 operational amplifier is not more than 40 μV [21]. Under higher correlation, the error of measurement increases and the output signal of the converter starts to converge to the power supply voltage of the operational amplifier.

**Figure 4:**
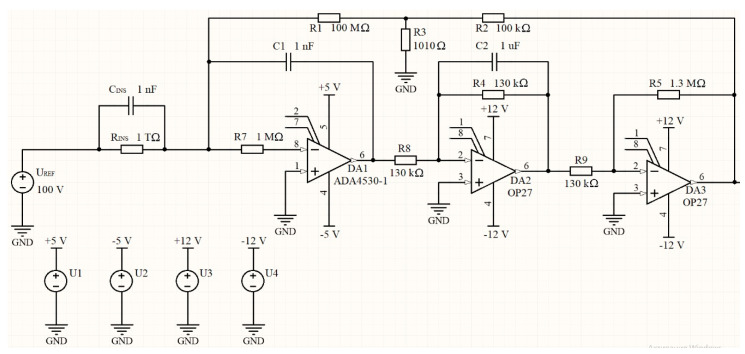
The electrical circuit of the resistance-to-voltage converter under study in the electrical circuit design software Altium Designer.

Figure 4 represents the electrical circuit of the resistance-to-voltage converter under study.

The converter comprises three cascades with the common negative feedback: amplifier integrator *DA*_1_, low-pass filter based on amplifier *DA*_2_, and direct-current amplifier *DA*_3_.

Amplifier transmission coefficient *DA*_3_ is described in the equation:

(5) $$K_{3}=-\frac{R_{5}}{R_{9}},$$

For the amplifier *DA*_3_ the coefficient *K*_3_ = −10 (*R*_9_ = 130 kΩ, *R*_5_ = 1.3 MΩ).

Frequency response for low-pass filter is expressed in the formula:

(6) $$K_{2}(j\cdot \omega )=-\frac{R_{4}}{R_{8}}\cdot \frac{1}{1+j\cdot \omega \cdot R_{4}\cdot C_{2}},$$

For the circuit discussed *R*_8_ = 130 kΩ, *R*_4_ = 130 kΩ, *C*_2_ = 1 μF.

Thus, the frequency response for the combined three cascaded resistance-to-voltage converters is described with the second-order equation:

(7) $$K(j\cdot \omega )=-\frac{R_{EQ}}{R_{INS}}\cdot \frac{1}{1+j\cdot \omega \cdot b_{1}+{\left(j\cdot \omega \right)}^{2}\cdot b_{2}},$$

where $b_{1}=C_{1}\cdot R_{EQ}\cdot \frac{R_{8}}{R_{4}}\cdot \frac{1}{K_{3}}$; $b_{2}=C_{1}\cdot R_{EQ}\cdot C_{2}\cdot R_{4}\cdot \frac{R_{8}}{R_{4}}\cdot \frac{1}{K_{3}}$.

For the operating current *J_X_*, set by resistance *R_INS_* under measurement, and the reference voltage *U_REF_*, frequency response will be expressed in a similar way, with the exception of resistance *R_INS_* which is not present in the formula:

(8) $$J_{X}(j\cdot \omega )=-R_{0}\cdot \frac{1}{1+j\cdot \omega \cdot b_{1}+{\left(j\cdot \omega \right)}^{2}\cdot b_{2}},$$

Coefficients of the frequency response of the teraohmmeter amplifier depend solely on the circuit parameters.

Under equivalent (reference) resistance *R_EQ_* = 10 GΩ, capacitance *C*_1_ = 1 nF and the parameters stated above of the circuit elements, the coefficients equal *b*_1_ = 1.0, *b*_2_ = 0.13. At these values, the coefficient of noise attenuation at the frequency of 50 Hz equals 82.16 dB, and the transient response is 2.7 s.

## 3. Results

### 3.1. Theoretical Studies of Fast Response Time of the Resistance-to-Voltage Converter with T-Shaped Feedback

To perform initial measurements of the fast response time of the resistance-to-voltage converter with T-shaped feedback, it is necessary to simulate the process of the measurement of insulation resistance for cable products in the electronic circuit design software Altium Designer.

As measurable values, it is necessary to take the reference characteristics of cables that are currently produced. Submarine coaxial cables possess the maximum acceptable insulation resistance (50 GΩ·km), in addition to relatively high electric capacitance (100 nF/km) (Table 2) [23,24,25,26,27]. Correspondingly, the time constant for such cables can reach up to 5000 s. This correlation of the values of insulation resistance and electrical capacitance of cable will be selected as maximum acceptable value.

Noise immunity and fast response time of the resistance-to-voltage converter presented in Figure 4 depends, first, on the value of capacitor *C*_1_ in the integrator. With insufficient capacitor *C*_1_ capacitance, the level of noise in the converter increases and the *DA*_1_ operational amplifier converges to saturation, which leads to non-operability of the converter. Thus, it is necessary to select the optimal value for the capacitor *C*_1_ capacitance that allows converter operability in conjunction with its maximum fast response time.

During experiments of actual submarine coaxial cables of different lengths *l*, it is easier to use simulation of resistance *R_INS_* and capacitance *C_INS_* by resistors and capacitors:

(1) *R_INS_* not less 500 GΩ and *C_INS_* less 10 nF when *l* = 100 m.
(2) *R_INS_* not less 50 GΩ and *C_INS_* less 100 nF when *l* = 1000 m.
(3) *R_INS_* not less 5 GΩ and *C_INS_* less 1000 nF when *l* = 10,000 m.

In all of the experiments the resistance of feedback was chosen in such a way that the output voltage *U_OUT_* within the measurable range was equal to 1 V. Resistance values of T-shaped feedback were chosen in accordance with the conclusions (recommendations) obtained in the work [20]. The transient response was understood as the time *T_S_*, after which the difference of the current value of transient response from the steady-state value of resistance was 5%.

**Figure 5:**
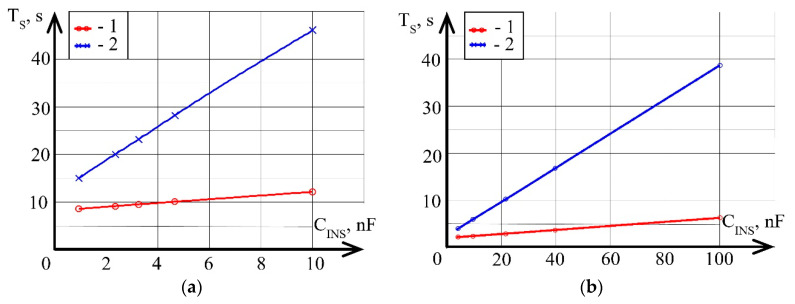
Theoretical dependences of the settling time during the measurements of insulation resistance on capacitance *C_INS_* (*C*_1_ = 1 nF, *U_REF_* = 100 V) (**a**) when *R_INS_* = 1 TΩ: 1—the resistance-to-voltage converter with T-shaped feedback with *R_EQ_* = 10 GΩ (*R*_1_ = 100 MΩ, *R_2_* = 100 kΩ, *R*_3_ = 1010 Ω); 2—the resistance-to-voltage converter with a classical feedback system with *R_EQ_* = 10 GΩ; (**b**) when *R_INS_* = 100 GΩ: 1—the resistance-to-voltage converter with T-shaped feedback with *R_EQ_* = 1 GΩ (*R*_1_ = 10 MΩ, *R*_2_ = 100 kΩ, *R*_3_ = 1010 Ω); 2—the resistance-to-voltage converter with a classical feedback system with *R_EQ_* = 1 GΩ.

**Figure 6:**
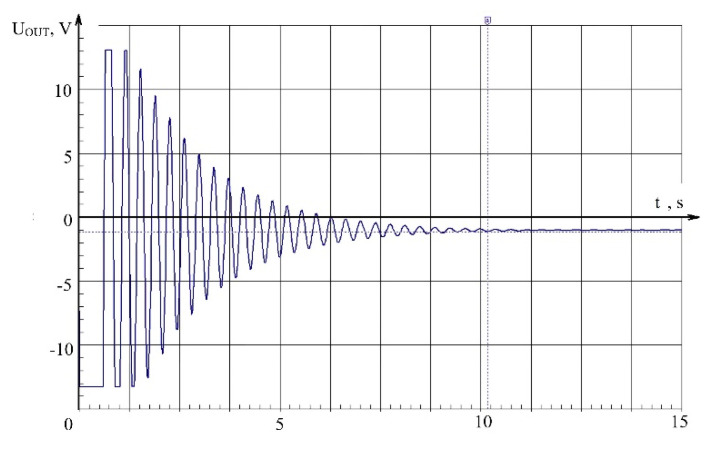
The transient response of the output voltage of the resistance-to-voltage converter with T-shaped feedback (*R*_1_ = 1 MΩ, *R*_2_ = 100 kΩ, *R*_3_ = 1010 Ω, *R_EQ_* = 0.1 GΩ, *R_INS_* = 10 GΩ, *C_INS_* = 100 nF, *C*_1_ = 1 nF, *U_REF_* = 100 V).

Figure 5 demonstrates the results of the simulation of dependence of the settling time of resistance under measurement on capacitance *C_INS_*, when *C*_1_ = 1 nF and measurable resistance is 1 TΩ and 100 GΩ. During measurements of resistance of 10 GΩ, the output voltage of the resistance-to-voltage converter was not set for the whole range of the capacitance *C_INS_* under study using the converter with a classical feedback system of the operational amplifier, whereas the converter with T-shaped feedback proved to be operable only with the capacitance *C_INS_* of not more than 100 nF. At this value, settling of the output voltage was performed with reregulation of the transient response of more than 100% (Figure 6). Based on this, it is possible to conclude that, in this configuration, the resistance-to-voltage converter possesses a small stability margin on the range of measurements of 10 GΩ. In the range of measurements of 100 GΩ, settling of the output voltage also occurred with reregulation of the transient response of more than 100%, however, a shorter time for reregulation was required, and, correspondingly, a greater stability margin was achieved. In the range of measurements of 1 TΩ, the settling of the output voltage was monotonous without reregulation.

Based on the obtained dependencies, the advantages of the resistance-to-voltage converter with T-shaped feedback are evident:

− the settling time depends to a lesser degree on the capacitive component of the object under control, and at the range of measurements of 1 TΩ it has practically a steady-state value (about 10 s) within the whole capacitance range under study;
− fast response time is 1.5–6 times better, depending on the range of measurements of resistance and capacitance value.

It is also worth mentioning that, using the resistance-to-voltage converter with this configuration, the settling time depends on the capacitance within the range under study, in compliance with the linear law.

**Figure 7:**
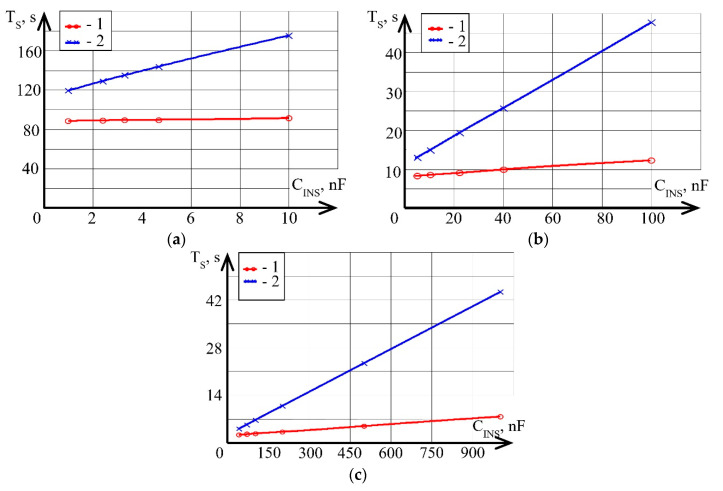
Theoretical dependences of the settling time during insulation resistance measurements on capacitance *C_INS_* (*C*_1_ = 10 nF, *U_REF_* = 100 V) (**a**) when *R_INS_* = 1 TΩ: 1—the resistance-to-voltage converter with T-shaped feedback with *R_EQ_* = 10 GΩ (*R*_1_ = 100 MΩ, *R*_2_ = 100 kΩ, *R*_3_ = 1010 Ω); 2—the resistance-to-voltage converter with a classical feedback system with *R_EQ_* = 10 GΩ; (**b**) when *R_INS_* = 100 GΩ: 1—the resistance-to-voltage converter with T-shaped feedback with *R_EQ_* = 1 GΩ (*R*_1_ = 10 MΩ, *R*_2_ = 100 kΩ, *R*_3_ = 1010 Ω); 2—the resistance-to-voltage converter with a classical feedback system with *R_EQ_* = 1 GΩ; (**c**) when *R_INS_* = 10 GΩ: 1—the resistance-to-voltage converter with T-shaped feedback with *R_EQ_* = 0.1 GΩ (*R*_1_ = 1 MΩ, *R*_2_ = 100 kΩ, *R*_3_ = 1010 Ω); 2—the resistance-to-voltage converter with a classical feedback system with *R_EQ_* = 0.1 GΩ.

Figure 7 demonstrates the simulation results of dependence of the settling time of resistance under measurement on capacitance *C_INS_*, when *C*_1_ = 10 nF.

The resistance-to-voltage converter was demonstrated to be operable within all the ranges of resistance measurements under study.

When measuring the resistance of 1 TΩ, the settling time of the output voltage was monotonous without reregulation. However, due to the increase in the integrator capacitance, the fast response time significantly decreased. In addition, the settling time for the resistance under measurement was more than 90 s for both variants of the resistance-to-voltage converters. This is not acceptable in accordance to [7], from which the reading is to take place within 60 s. The characteristic of dependences was linear.

At the range of measurements of 100 GΩ, the settling of the output voltage was performed monotonously without reregulating. The fast response time did not change significantly for the resistance-to-voltage converter with a classical feedback system or the resistance-to-voltage converter with T-shaped feedback. The characteristic of dependences was linear.

At the range of measurements of 10 GΩ, the settling of the output voltage was performed with reregulation of the transient response of more than 100%. The fast response time of the resistance-to-voltage converter with T-shaped feedback was demonstrated to be 1.5–6 times better than that of the resistance-to-voltage converter with a classical feedback system.

**Figure 8:**
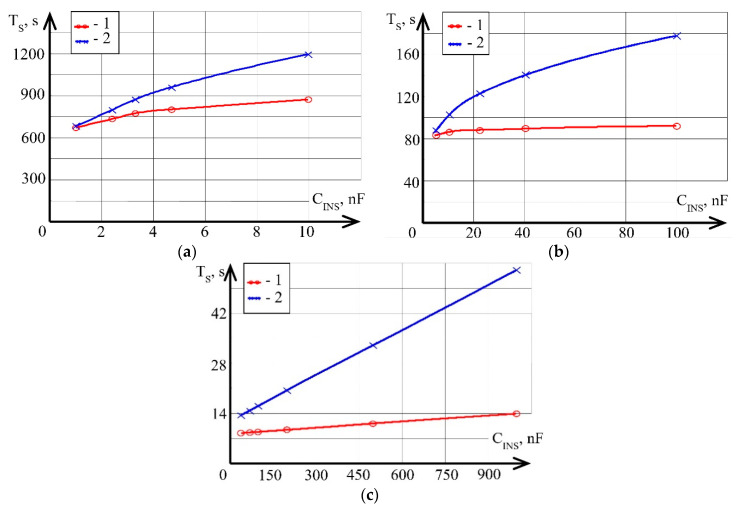
Theoretical dependencies of the settling time during insulation resistance measurements on capacitance *C_INS_* (*C*_1_ = 100 nF, *U_REF_* = 100 V) (**a**) when *R_INS_* = 1 TΩ: 1—the resistance-to-voltage converter with T-shaped feedback with *R_EQ_* = 10 GΩ (*R*_1_ = 100 MΩ, *R*_2_ = 100 kΩ, *R*_3_ = 1010 Ω); 2—the resistance-to-voltage converter with a classical feedback system with *R_EQ_* = 10 GΩ; (**b**) when *R_INS_* = 100 GΩ: 1—the resistance-to-voltage converter with T-shaped feedback with *R_EQ_* = 1 GΩ (*R*_1_ = 10 MΩ, *R*_2_ = 100 kΩ, *R*_3_ = 1010 Ω); 2—the resistance-to-voltage converter with a classical feedback system with *R_EQ_* = 1 GΩ; (**c**) when *R_INS_* = 10 GΩ: 1—the resistance-to-voltage converter with T-shaped feedback with *R_EQ_* = 0.1 GΩ (*R*_1_ = 1 MΩ, *R*_2_ = 100 kΩ, *R*_3_ = 1010 Ω); 2—the resistance-to-voltage converter with a classical feedback system with *R_EQ_* = 0.1 GΩ.

With increase in the capacitance value *C_1_* in the integrator up to 100 nF, the settling time of the resistance under measurement (1 TΩ and 100 GΩ) on the capacitance increased to 60 s. However, during the resistance measurements of 10 GΩ, the settling time was no more than 14 s for the resistance-to-voltage converter with T-shaped feedback and no more than 55 s for the resistance-to-voltage converter with a classical feedback system (Figure 8).

### 3.2. Experimental Studies of Fast Response Time of the Resistance-to-Voltage Converter with T-Shaped Feedback

Experimental studies were conducted to verify the results obtained during the simulation of the data on the fast response time of the resistance-to-voltage converter with T-shaped feedback when monitoring large resistance values which possess a capacitive component.

In the experimental set-up, the assembly of the elements of the resistance-to-voltage converter was performed on a polytetrafluorethylene plate. This material was chosen due to its great resistivity (about 10^18^ Ω·m), which allowed minimization of leakage currents [28].

**Figure 9:**
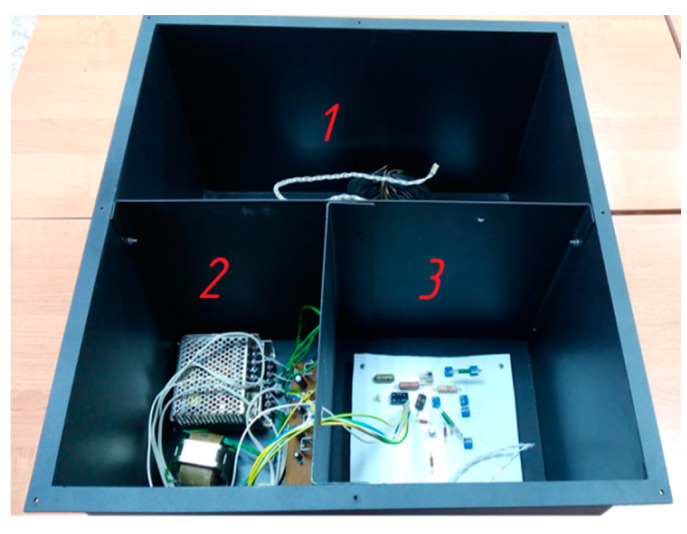
The shielding cell: 1—cell designated for cable allocation; 2—power supply; 3—the resistance-to-voltage converter.

All studies were performed in a shielding cell (Figure 9), which allowed for minimization of the impact of external noise and fair treatment of the obtained experimental results.

#### 3.2.1. The Usage of the Simplified Circuit of the Cable Substitution as the Object of Measurements

**Figure 10:**
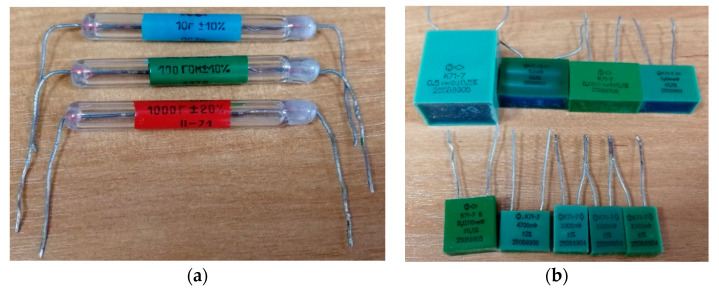
Appearance of (**a**) KVM type resistor (**b**) K71-7 type capacitor.

Simulation of the insulation material with the determined properties was performed by simultaneously connecting the KVM resistor (resistor type: composite, vacuum) and K71-7 capacitor (polypropylene film, metalized, single-layer) (Figure 10) [29,30]. Their specifications are given in Table 3.

As can be seen from Table 3, the stated insulation resistance of the K71-7 lead–lead capacitor is not less than 50 GΩ, which is comparable with nominal values of the resistances *R_INS_* under study and can impact the measurement results. It is worth mentioning that the specifications for capacitors state the minimum acceptable insulation resistance, while in practice it is significantly greater. During the experiments, comparison of the steady-state value of the output voltage of the resistance-to-voltage converter was undertaken when measuring *R_INS_* resistance with and without connecting *C_INS_*. The data of the output voltage of the resistance-to-voltage converter was identical. This means that the insulation resistance of the K71-7 lead–lead capacitor is an order of magnitude greater than 1 TΩ.

The values of the resistance and capacitances were chosen to be analogous to the theoretical studies. The basic electronic circuit of the resistance-to-voltage converter is presented in Figure 4.

Measurements of the settling time of the transient response of the output voltage of the resistance-to-voltage converter were performed with a digital oscilloscope ADS-5304, which allowed long-term data recording to be undertaken. The detailed specifications for the oscilloscope are provided in the work [31].

**Figure 11:**
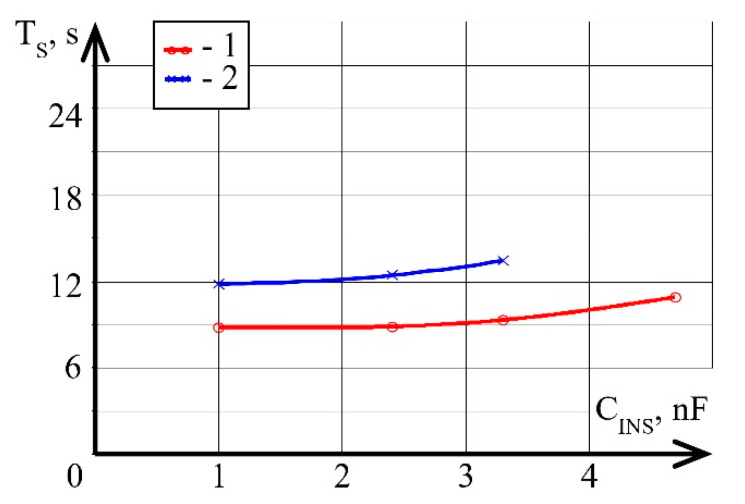
Experimental dependencies of the settling time during insulation resistance measurements on capacitance *C_INS_* (*C_1_* = 1 nF, *U_REF_* = 100 V) when *R_INS_* = 1 TΩ: 1—the resistance-to-voltage converter with T-shaped feedback with *R_EQ_* = 10 GΩ (*R_1_* = 100 MΩ, *R_2_* = 100 kΩ, *R_3_* = 1010 Ω); 2—the resistance-to-voltage converter with a classical feedback system with *R_EQ_* = 10 GΩ.

**Figure 12:**
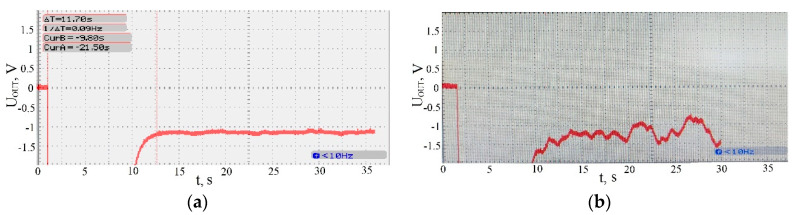
Oscillograms of the transient response of the output voltage of the resistance-to-voltage converter (*U_REF_* = 100 V, *R_EQ_* = 10 GΩ, *R_INS_* = 1 TΩ, *C_INS_* = 10 nF, *C_1_* = 1 nF): (**a**) with T-shaped feedback (*R_1_* = 100 MΩ, *R_2_* = 100 kΩ, *R_3_* = 1010 Ω); (**b**) with a classical feedback system.

Figure 11 demonstrates the experimental dependences of the settling time of resistance under measurement on capacitance *C_INS_*, when *C*_1_ = 1 nF, and resistance under measurement of 1 TΩ. When *C_INS_* exceeds 3.3 nF for the resistance-to-voltage converter with a classical feedback system, and when *C_INS_* exceeds 4.7 nF for the resistance-to-voltage converter with T-shaped feedback, due to insufficient capacitance in the integrator of the converter, even under monotonous settling of the transient response of output voltage, spontaneous signal steps appear that do not attenuate at a later time and significantly increase the error of measurement (Figure 12). This effect is caused by insufficient noise immunity of the converter and was not apparent during simulation with analogous characteristics of electronic components.

At lower ranges of measurement (10 and 100 GΩ), the converter in this configuration proved to be inoperative. The capacitance *C_1_* value was increased to solve this problem.

**Figure 13:**
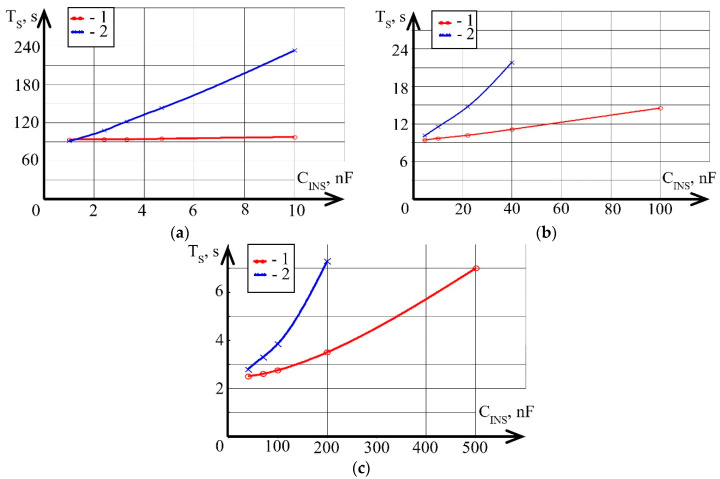
Experimental dependencies of the settling time during insulation resistance measurements on capacitance *C_INS_* (*C_1_* = 10 nF, *U_REF_* = 100 V) (**a**) when *R_INS_* = 1 TΩ: 1—the resistance-to-voltage converter with T-shaped feedback with *R_EQ_* = 10 GΩ (*R_1_* = 100 MΩ, *R_2_* = 100 kΩ, *R_3_* = 1010 Ω); 2—the resistance-to-voltage converter with a classical feedback system with *R_EQ_* = 10 GΩ; (**b**) when *R_INS_* = 100 GΩ: 1—the resistance-to-voltage converter with T-shaped feedback with *R_EQ_* = 1 GΩ (*R_1_* = 10 MΩ, *R_2_* = 100 kΩ, *R_3_* = 1010 Ω); 2—the resistance-to-voltage converter with a classical feedback system with *R_EQ_* = 1 GΩ; (**c**) when *R_INS_* = 10 GΩ: 1—the resistance-to-voltage converter with T-shaped feedback with *R_EQ_* = 0.1 GΩ (*R_1_* = 1 MΩ, *R_2_* = 100 kΩ, *R_3_* = 1010 Ω); 2—the resistance-to-voltage converter with a classical feedback system with *R_EQ_* = 0.1 GΩ.

Figure 13 demonstrates the experimental dependences of the settling time of resistance under measurement on capacitance *C_INS_* when *C_1_* = 10 nF.

At the range of measurements of resistance of 100 GΩ and 1 TΩ, the settling of the output voltage was performed monotonously without reregulating. However, during measurements of 1 TΩ, the settling time was more than 90 s. In the resistance-to-voltage converter with a classical feedback system when *R_INS_* = 100 GΩ and *C_INS_* exceeds 40 nF, spontaneous signal steps were observed, as demonstrated in Figure 12.

When *R_INS_* = 10 GΩ, the settling of the output voltage was performed with reregulation of the transient response of more than 100%. When *C_INS_* exceeds 200 nF for the resistance-to-voltage converter with a classical feedback system, and when *C_INS_* exceeds 500 nF for the resistance-to-voltage converter with T-shaped feedback, spontaneous signal steps were also observed that led to the non-operability of the converter.

**Figure 14:**
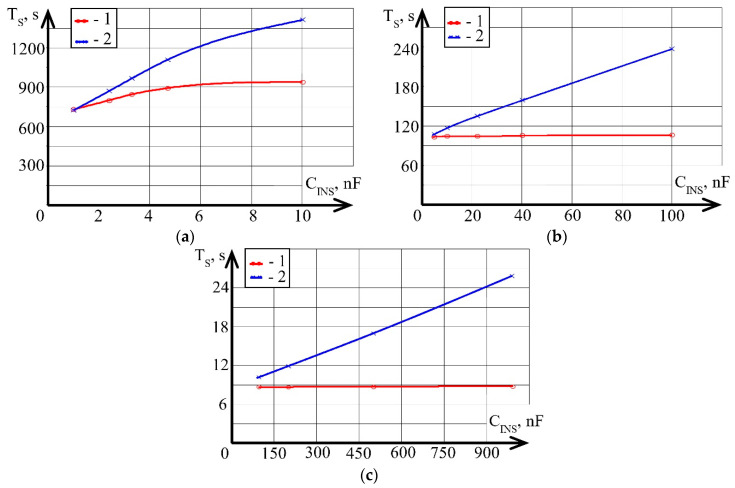
Experimental dependencies of the settling time during insulation resistance measurements on capacitance *C_INS_* (*C_1_* = 100 nF, *U_REF_* = 100 V) (**a**) when *R_INS_* = 1 TΩ: 1—the resistance-to-voltage converter with T-shaped feedback with *R_EQ_* = 10 GΩ (*R*_1_ = 100 MΩ, *R*_2_ = 100 kΩ, *R*_3_ = 1010 Ω); 2—the resistance-to-voltage converter with a classical feedback system with *R_EQ_* = 10 GΩ; (**b**) when *R_INS_* = 100 GΩ: 1—the resistance-to-voltage converter with T-shaped feedback with *R_EQ_* = 1 GΩ (*R*_1_ = 10 MΩ, *R*_2_ = 100 kΩ, *R*_3_ =1010 Ω); 2—the resistance-to-voltage converter with a classical feedback system with *R_EQ_*=1 GΩ; (**c**) when *R_INS_* = 10 GΩ: 1—the resistance-to-voltage converter with T-shaped feedback with *R_EQ_* = 0.1 GΩ (*R*_1_ = 1 MΩ, *R*_2_ = 100 kΩ, *R*_3_ = 1010 Ω); 2—the resistance-to-voltage converter with a classical feedback system with *R_EQ_* = 0.1 GΩ.

Figure 14 demonstrates the experimental dependences of the settling time of resistance under measurement on capacitance *C_INS_*, when *C_1_* = 100 nF.

At all of the ranges of measurements of resistance under study, the settling of the output voltage was performed monotonously without reregulating.

Analogously to simulation, during resistance measurements of 1 TΩ, the inertia of the resistance-to-voltage converter significantly increased and the settling time of the output voltage required more than 12 min, which is not acceptable.

At the ranges of measurements of 10 and 100 GΩ, the characteristics of dependences of the settling time on the capacitance were close to linear; these were constant and did not depend on changes of the capacitive component for the converter with T-shaped feedback within the range under study *C_INS_*. The resistance-to-voltage converter with T-shaped feedback demonstrated a better fast response time.

#### 3.2.2. The Usage of the Real Cable as the Object of Measurement

**Figure 15:**
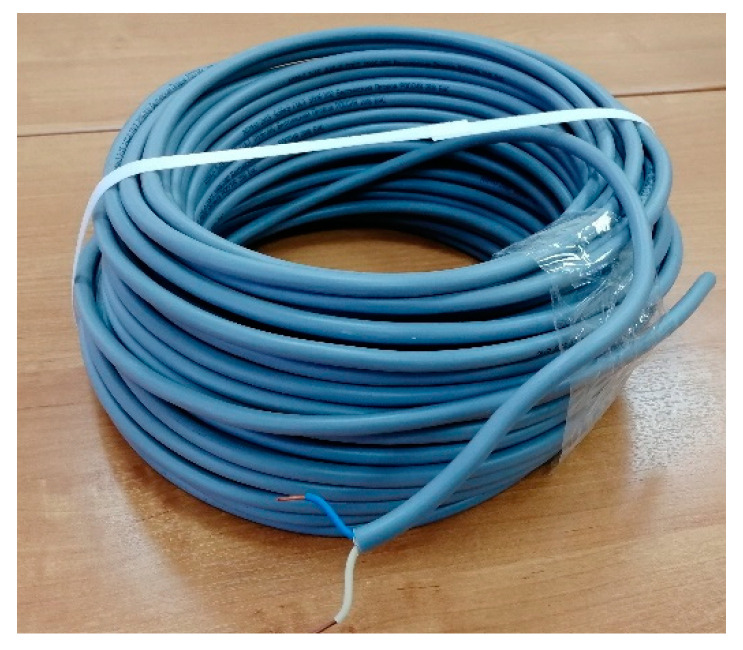
PVC insulated and sheathed cable NYM-O 2 × 1.5.

PVC insulated and sheathed cable NYM-O 2 × 1.5 was chosen as the object of measurements (Figure 15).

The cable possesses the following technical characteristics: length *l* = 50 m, insulation resistance *R_INS_* not less than 400 MΩ, and insulation capacitance *C_INS_* not exceeding 10 nF.

**Figure 16:**
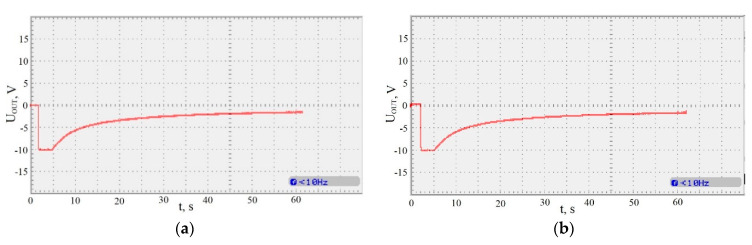
Oscillograms of the transient response of the output voltage of the resistance-to-voltage converter (*U_REF_* = 100 V, *R_EQ_* = 100 MΩ, *C*_1_ = 100 nF): (**a**) with T-shaped feedback (*R*_1_ = 1 MΩ, *R*_2_ = 100 kΩ, *R*_3_ = 1010 Ω); (**b**) with a classical feedback system.

The measurements were conducted in compliance with the standard [7], that is, the readings of the obtained resistance were performed after 60 s of applying the reference voltage *U_REF_* to the cable. Figure 16 shows the oscillograms.

Based on the analysis of the obtained oscillograms, it was concluded that the transition processes for the converter with T-shaped feedback and for the converter with a classical feedback system are the same and in agreement with the results of theoretical modeling.

The obtained equal fast response time of the converters is explained by the very low value of the cable insulation capacitance. In Figure 14c, this corresponds to the initial line at which the effect of the fast response time is not yet significant.

The output voltage of the converters *U_OUT_*, 60 s after using the reference voltage *U_REF_*, was −1.8 V. According to Formula (1), the obtained cable insulation resistance was calculated and found to be equal to 5.55 GΩ, which significantly increases its certified value.

## 4. Conclusions

From a comparison of the results of simulation with the data obtained during the experiments the following conclusions can be made:

- The characteristics of the dependencies of the settling time of the resistance under measurement on capacitance are similar to the analogous characteristics of electronic components of the resistance-to-voltage converter.
- The fast response time of the resistance-to-voltage converter with T-shaped feedback is better than that of the resistance-to-voltage converter with a classical feedback system.
- The simulation results of the fast response time of the resistance-to-voltage converter were experimentally verified. Insignificant differences of experimental results from the simulation data are caused by large departures of the components *R_INS_* and *C_INS_* from the nominal values.
- With minor capacitance of the object of measurement, there are no advantages in the fast response time of the resistance-to-voltage converter with T-shaped feedback in comparison to the resistance-to-voltage converter with the classical feedback system.
- There is no optimal value for the capacitance of the *C_1_* capacitor, for all the ranges of measurements of insulation resistance for cable products under study, simultaneously. To provide for the converter operability combined with maximum fast response time, it is necessary to change the capacitance of *C_1_* capacitor and the range of measurement:
  (1) when *R_EQ_* = 0.1 GΩ, *C*_1_ = 100 nF;
  (2) when *R_EQ_* = 1 GΩ, *C*_1_ = 10 nF;
  (3) when *R_EQ_* = 10 GΩ, *C*_1_ = 1 nF.

It was experimentally proven that, with the usage of the simplified circuit of the cable substitution, the resistance-to-voltage converter with T-shaped feedback possesses a fast response time that is 1–3 times faster than that of the resistance-to-voltage converter with a classical feedback system.

To obtain more objective results on the fast response time of resistance-to-voltage converters during measurements of cables’ insulation resistance, further investigation is needed to be performed on the premises of cable manufacturing plants to enable access to large coils of cables.

## Figures and Tables

**Table 1:** The main characteristics of operational amplifiers with low input current.

Operational Amplifier	*I_IN_*, fA ^1^	*U_OF_*, μV ^2^	*R_IN_*, TΩ ^3^	*C_IN_*, pF ^4^	*U_SUP_*, V ^5^	*GBP,* MHz ^6^	***A_D_*^7^**
AD549L	±60	±500	1000	0,8	±15	1	˃0.1 × 10^6^
ADA4530-1	±20	±40	˃100	8	±8	2	˃3.16 × 10^6^
LTC6268	±20	±700	˃1	0,1	+5	4000	˃0.125 × 10^6^
OPA128LM	±75	±500	1000	2	+15	1	˃3.16 × 10^5^
LMP7721	±20	±150	˃1	15	+5	15	˃40 × 10^3^

^1^ Input current of operational amplifier; ^2^ Offset voltage of operational amplifier; ^3^ Input resistance of operational amplifier; ^4^ Input capacity of operational amplifier; ^5^ Power supply voltage of operational amplifier; ^6^ Gain bandwidth product; ^7^ Open loop gain.

**Table 2:** Reference characteristics of cables.

Cable Type	*R_INS_* Not Less, GΩ·km	*C_INS_* Less, nF/km	Nominal Voltage, kV
Submarine coaxial cable (PE core insulation, PE outer sheath)	50	100	3.5
Instrumentation cable (polyethylene (PE) core insulation, pairs individually foiled, Polyvinyl chloride (PVC) outer sheath)	2	370	0.3
Instrumentation cable (PVC, overall screened, unarmored)	25	450	0.3/0.5
Data transmission cable (PE core insulation, PVC outer sheath)	10	52	0.15
Cable for drag chains, halogen-free	0.1	60	0.3
Cross-linked polyethylene (XLPE) insulated and PVC sheathed power cables	0.1	800	0.6/1.0
Intrinsically safe cables (Sheathing PVC of high oxygen index is UV radiation and weather resistant, is self-extinguishing and flame retardant)	0.02	140	0.6/1.0
Local area network cables (PE core insulation, PVC outer sheath)	5	50	0.15
Coaxial television cable (PE core insulation, PVC outer sheath)	10	67	3.5
PVC insulated and sheathed cables	0.02	200	0.6/1.0

**Table 3:** The specifications of KVM resistor and K71-7 capacitor.

Specification Name	K71-7	KVM
Nominal voltage	250 V	100 V
Insulation resistance lead–lead, not less	50 GΩ	-
Insulation resistance lead–sheath, not less	70 GΩ	-
Loss tangent, not exceeding	0.001	-
Permissible error of nominal value	±0.5%, ±1%, ±2%	±10%, ±20%

## Data Availability

Data sharing not applicable.
